# Textbook oncologic outcomes in colorectal cancer surgery: a systematic review

**DOI:** 10.3389/fonc.2025.1474008

**Published:** 2025-05-26

**Authors:** Giang Son Arrighini, Alessandro Martinino, Victoria Zecchin Ferrara, Laura Lorenzon, Francesco Giovinazzo

**Affiliations:** ^1^ Faculty of Medicine and Surgery, University of Bologna, Bologna, Italy; ^2^ Department of Surgery, Duke University, Durham, NC, United States; ^3^ Faculty of Medicine and Surgery, University of Padua, Padua, Italy; ^4^ Fondazione Policlinico Universitario Agostino Gemelli Istituto di Ricovero e Cura a Carattere Scientifico (IRCCS), Catholic University of the Sacred Heart, Rome, Italy; ^5^ Department of Surgery, UniCamillus-Saint Camillus International University of Health Sciences, Rome, Italy; ^6^ Department of Surgery, Saint Camillus Hospital, Treviso, Italy

**Keywords:** colorectal surgery, colorectal cancer, colon cancer, textbook outcome, textbook oncologic outcome, surgical quality

## Abstract

**Introduction:**

The concept of “textbook outcome” has been updated to encompass the principles of surgical oncology and the related outcomes [textbook oncologic outcome (TOO)]. This systematic review aims to synthesize the numerous definitions of TOO in the context of colorectal surgery. The goal is to promote the development of a definition that has universal recognition and worldwide acceptability, hence improving surgical quality standards and patient outcomes.

**Methods:**

A systematic literature review was conducted using PRISMA guidelines. The databases PubMed, Web of Science, and Scopus were searched for studies that addressed TOO in colorectal cancer surgeries. The database search was conducted on 30 April 2024, and the primary study’s quality was assessed using the Newcastle–Ottawa Scale.

**Results:**

A total of 13 studies were included. Common TOO parameters included radical resection, lymph node (LN) yield ≥12, no Clavien-Dindo grade ≥III complications, length of stay (75th percentile), no 30-day readmissions, and no 30-day mortality. Factors influencing TOO achievement included surgical risk, gender, tumor stage, and socioeconomic factors. Patients achieving TOO showed better long-term survival. Variability in TOO definitions highlighted the need for standardization.

**Conclusion:**

TOO is an effective indicator for evaluating the quality of colorectal cancer surgery. It provides a comprehensive evaluation of surgical outcomes, which helps in guiding patient decisions and measuring hospital performance. By standardizing the parameters of TOO, the consistency and quality of care across different institutions can be improved. We propose a unified definition of TOO for colorectal cancer surgery: radical resection, LN yield ≥12, no Clavien-Dindo grade ≥III complications, length of stay (75th percentile), no 30-day readmissions, and no 30-day mortality.

## Introduction

Colon cancer is still one of the most common types of cancer, contributing considerably to the global increase in cancer-related deaths. Despite advancements in multimodal treatments that have enhanced patient outcomes, the disease continues to pose a substantial health challenge (1, 2). The quality of oncologic surgery has historically been assessed using a range of metrics, including postoperative mortality and morbidity, lymph node (LN) yield, reoperation rates, readmission rates, and cancer-related survival. These days, the evaluation of care quality has become more and more important (3, 4), as research indicates that patients are prepared to go further to receive higher quality care and prefer to select their treatment hospital based on its result statistics (5, 6).

A proposed composite quality score known as “textbook outcome” (TO) represents the optimal “textbook” hospitalization for complicated surgical operations by integrating multiple postoperative endpoints (7, 8). TO is the percentage of patients who receive ideal surgical care and for whom all intended short-term goals of care are achieved. Notably, TO extends beyond mere event recording to underscore the disparities in performance across medical institutions. This distinctive feature elevates TO as a potent instrument for hospital comparison, enabling the identification of exemplary practices that might set a standard for excellence (7, 9). Based on the TO framework, the textbook oncologic outcome (TOO) concept is a composite outcome measure that is attained after an oncological operation when all desired quality criteria are satisfied (10). Achievement of TOO has been demonstrated to be linked to increased long-term survival across a range of malignancies, including colon and rectal cancers, underscoring its clinical usefulness as a criterion for surgical cancer treatment quality (11).

This systematic review is designed to summarize the various definitions of TOO within the contexts of colon and rectal cancer surgeries. The aim is to foster the establishment of a definition that gains widespread recognition and international acceptance. By achieving a uniform understanding and application of TOO, this effort seeks to enhance the benchmarking of surgical quality and improve patient outcomes in this medical domain.

## Methods

### PICO process and search strategy

“In *patients undergoing colorectal surgery* (P), does *TOO* (I) compared to *traditional quality metrics*, like postoperative mortality and morbidity (C), influence *a comprehensive set of primary and secondary outcomes* (O) reflecting the multifaceted nature of colorectal cancer surgery?

A systematic literature review was carried out in accordance with PRISMA guidelines to deepen comprehension of the topic and offer valuable perspectives to the medical field. Our research involved searching PubMed, Web of Science, and Scopus databases. We utilized the search terms “colon cancer”, “rectal cancer”, “colorectal”, “textbook outcome”, and “TOO”. Additionally, we identified articles from the references of the retrieved publications. The date of the search was 30 April 2024.

### Inclusion and exclusion criteria

All English language studies that addressed TOO in colorectal cancer surgery were included. Non-English language studies, no full-text available studies, case series, and case reports were excluded from our analysis.

### Study selection, data extraction, and quality assessment

Two researchers (G.S.A. and A.M.) independently evaluated study titles and abstracts using predefined search parameters to select studies that met the entry criteria. The same two researchers (G.S.A. and A.M.) assessed the complete texts for inclusion and gathered data. In cases of discrepancy, a third reviewer was consulted (V.Z.F.). G.S.A. and V.Z.F. subsequently examined all selected articles and collected the data using Excel(R).

For each included article, general study characteristics such as study design, year, country, sample size, and database used were extracted, along with all reported parameters used to define TOO. These parameters included radical resection, LN yield ≥12, Clavien-Dindo complications, 30- or 90-day mortality, 30- or 90-day readmissions, length of hospital stay, reintervention, ostomy, and additional outcome measures such as conversion to open surgery, discharge destination, colonoscopy timing, surgery within 6 weeks, and receipt of adjuvant chemotherapy. Patient-related factors and predictors of TOO achievement, such as age, sex, cancer stage, surgical approach, and socioeconomic determinants, were also recorded. The Newcastle–Ottawa scale was used to evaluate the studies’ quality. 0–2 was regarded as low quality, 3–5 as acceptable, and 6–9 as good or outstanding. Not a single study was an RCT. We decided against conducting a meta-analysis due to the heterogeneity in outcome reporting and variations in study populations and methodologies.

## Results

**Figure 1:**
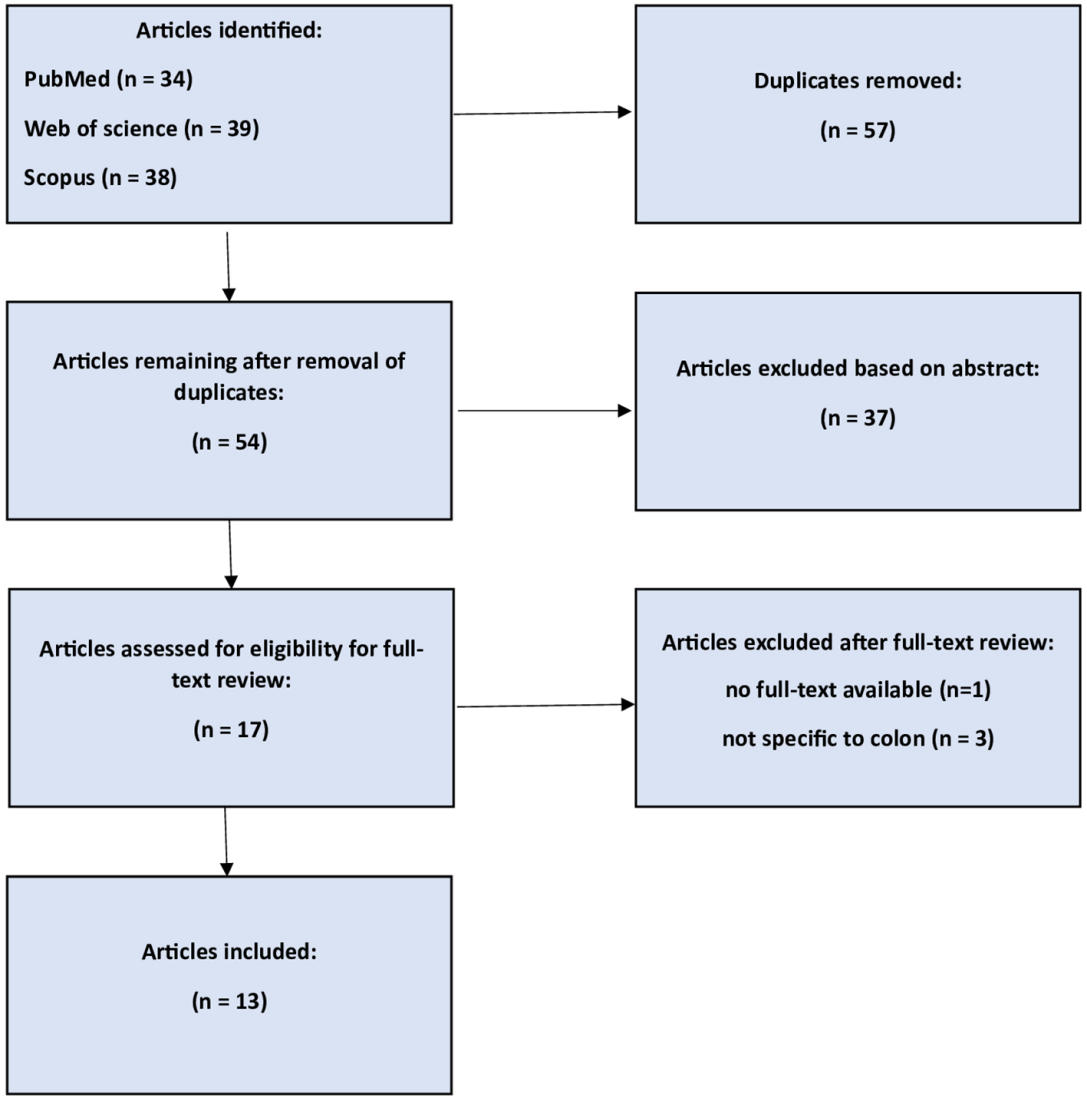
PRISMA flow diagram.

Following the initial search, 111 articles were collected. After eliminating duplicates and conducting a screening of titles and abstracts, we identified 17 articles published by April 2024 for inclusion. Four of these articles were removed from consideration for the reasons listed below: (1) full text was not available, 3 were not related to colon surgery; thus, 13 studies were suitable for review (**Figure 1**). In terms of the subjects covered, eight studies discussed TOO in colon cancer surgery, (2) TOO in colorectal cancer with a distinction between colon and rectum patients, while (3) studies did not have a distinction between colon and rectum patients. **Table 1** displays the studies’ characteristics, and **Supplementary Table S1** reports the studies’ quality assessment.

**Table 1:** Colon and colorectal cancer surgery textbook oncologic outcomes.

Colon cancer surgery	Year	Scope
Textbook outcome in colon carcinoma: implications for overall survival and disease-free survival.	2023	Definition of TOO.
The association between the composite quality measure “textbook outcome” and long-term survival in operated colon cancer.	2020	Definition of TOO.
Achieving a Textbook Outcome in Colon Cancer Surgery Is Associated with Improved Long-Term Survival.	2023	Definition of TOO.
Focusing on desired outcomes of care after colon cancer resections; hospital variations in “textbook outcome.”	2013	Definition of TOO.
A Novel Machine Learning Approach to Predict Textbook Outcome in Colectomy.	2024	Definition of TOO.
Assessment of Cancer Center Variation in Textbook Oncologic Outcomes Following Colectomy for Adenocarcinoma	2021	Definition of TOO.
Identifying best performing hospitals in colorectal cancer care; is it possible?	2020	Definition of TOO.

Colorectal cancer surgery (with distinction between colon and rectal surgery)	Year	Scope
Frailty assessment can predict textbook outcomes in senior adults after minimally invasive colorectal cancer surgery.	2023	Definition of TOO.
Textbook Oncological Outcomes for Robotic Colorectal Cancer Resections: An Observational Study of Five Robotic Colorectal Units.	2023	Definition of TOO.
Impact of safety‐net hospital burden on achievement of textbook oncologic outcomes following resection in for Stages I–IV colorectal cancer.	2024	Definition of TOO.

Colorectal cancer surgery (with no distinction between colon and rectal surgery)	Year	Scope
Textbook outcome contributes to long-term prognosis in elderly colorectal cancer patients.	2023	Definition of TOO.
Association between the environmental quality index and textbook outcomes among Medicare beneficiaries undergoing surgery for colorectal cancer (CRC).	2023	Definition of TOO.
The Association of Food Insecurity and Surgical Outcomes Among Patients Undergoing Surgery for Colorectal Cancer.	2023	Definition of TOO.

### Colon cancer surgery and TOO

Our comprehensive investigation revealed seven papers that examine the TOO in colon cancer surgery (**Table 2**). The study included 205,877 patients who underwent colon cancer surgery and were registered for TOO. A total of 124,420 patients achieved TOO, while 81,457 did not. Of the seven papers, one was published in 2024 (12), two were published in 2023 (13, 14), one in 2021 (15), one in 2020 (9), and one in 2013 (7); three of them were single-center studies (9, 13, 14) while four were multicenter studies (7, 12, 15, 16). Most of the patients come from a multicenter study based on the National Cancer Database (170,120 patients) (15).

**Table 2:** Colon surgery textbook outcomes.

Article (setting, period)	Rubio García JJ et al. (Single center, 2012-2016)	Yang CC et al. (Single center, 2010-2014)	Manatakis DK et al. (Single center, 2010-2020)	Kolfschoten NE et al. (Multicenter, 2010)	Ashraf Ganjouei A et al. (Multicenter, 2014-2020)	Sweigert PJ et al (Multicenter, 2010-2015)	Van Groningen JT et al. (Multicenter, 2013-2015)
Patients	*Total*: 564 *Achieved TOO*: 281(50%) *Not achieved TOO*: 283	*Total*: 804 *Achieved TOO*: 478(59%) *Not achieved TOO*: 326	*Total*: 128 *Achieved TOO*: 77(60%) *Not achieved TOO*: 51	*Total: 5582 Achieved TOO: 2735*(49%) *Not achieved TOO: 2847*	*Total: 20,498 Achieved TOO: 13,532*(66%) *Not achieved TOO: 6,966*	*Total: 170,120 Achieved TOO: 93,204*(55%) *Not achieved TOO: 76,916*	*Total: 8181 Achieved TOO: 5113*(62%) *Not achieved TOO: 3068*
TO variable							
Radical resection	yes	yes	yes	yes	no	yes	yes
Lymph node (LN) yield ≥ 12	yes	yes	yes	no	no	yes	no
No Clavien-Dindo grade ≥ III or ≥ II complications in the first 30 days	yes (≥ III)	no	yes (≥ II)	no	no	no	no
Hospital stay < 14 days	yes	no	yes (75th percentile of the study population: ≤ 11 days)	yes	yes (≤ 5 days)	Yes (≤ 75th percentile by year and operative approach)	yes
No 30-day readmission	yes	yes	yes	no	yes	yes	no
No 30-day mortality	yes	no	yes	yes	yes	yes	yes
No ostomy	no	yes	no	yes	no	no	yes
No reintervention	no	yes (no 30-day reintervention)	yes	yes	yes (no 30-day reintervention)	no	yes
Colonoscopy before/after surgery within 6 months	no	yes	no	no	no	no	No
Met the mentioned healthcare parameters within 6 weeks	no	yes	no	no	no	no	no
No unplanned stoma	no	no	yes	no	no	no	no
No adverse outcome within 30 days.	no	no	no	yes (within 30 days)	no	no	yes
No postoperative complications	no	no	no	no	yes	no	yes
Receipt of stage-appropriate adjuvant chemotherapy	no	no	no	no	no	yes	no

All the publications, except one (12), included radical resection as a TOO parameter (7, 9, 13–16). Four studies examined the number of LNs with a threshold of ≥12 (9, 13–15). One study included no Clavien-Dindo grade ≥III complications in the first 30 days as a TOO variable (13); one study included no Clavien-Dindo grade ≥II complications in the first 30 days (14).

Length of stay (LOS) was included in six studies: three studies mentioned a stay of <14 days (7, 13, 16), one study a stay of ≤5 days (12), one study a stay of ≤11 days. In one study, the median LOS varied according to the year and method of surgery, from 6 days for an open colectomy in 2010 to 4 days for a robot-assisted colectomy in 2015 (15).

Five studies mentioned no readmission in the first 30 days as a TOO parameter (9, 12–15). Six studies included no 30-day mortality as a TOO parameter (7, 12–16). No ostomy was considered in three studies (7, 9, 16) and in one study, no unplanned ostomy was considered a TOO variable (14). Receipt of stage-appropriate adjuvant chemotherapy was included in one study (15). Non-reintervention was included in three studies without specifying the time point (7, 14, 16) and two studies specified within 30 days (9, 12).

Some studies mentioned unique TOO parameters: no adverse outcome without a specific time point (16) and within 30 days (7), colonoscopy before/after surgery within 6 months (9), meeting all TOO parameters within 6 weeks (9), and no postoperative complications (12).

Overall, the most frequently included parameters were radical resection, LN yield ≥ 12, LOS, no 30-day mortality, no 30-day readmissions, no ostomy, and no reintervention.

### Colorectal cancer surgery with distinction between colon and rectum patients and TOO

We identified three papers that examine textbook outcome in colorectal cancer surgery with the distinction between colon and rectum patients (**Table 3**). The study includes 488,117 patients. Total colon cancer patients: 367,975; achieved TOO for colon surgery: 255,815; not achieved TOO for colon surgery: 112,160. Total rectal cancer patients: 84,922; achieved TOO for rectal surgery: 46,287; not achieved TOO for rectal surgery: 38,635. Total rectosigmoid junction cancer patients: 35,220; achieved TOO for rectosigmoid junction surgery: 23,376; not achieved TOO for rectosigmoid junction surgery: 11,844. Two papers were published in 2023 (17, 18) and one in 2024 (11). Most of the patients come from a multicenter study based on the National Cancer Database (487,195 patients) (11).

**Table 3:** Colorectal surgery (with distinction between colon and rectum patients) textbook outcomes.

Article (setting, period)	Azevedo JM et al. (Multicenter, 2012-2022)	Taffurelli G et al. (Single center, 2017-2021)	Wong P et al. (Multicenter, 2010-2019)
Patients	*-Total CC patients: 104 Achieved TOO for CS: 84(81%) Not achieved TOO for CS: 20 -Total RC patients: 397 Achieved TOO for RS:304(77%) Not achieved TOO for RS:93*	*-Total CC patients:316 Achieved TOO for CS: 217(69%) Not achieved TOO for CS: 99 -Total RC patients: 105 Achieved TOO for RS:72(73%) fot achieved TOO for RS:33*	*-Total CC patients:367,555 Achieved TOO for CS: 255,514(70%) Not achieved TOO for CS: 112,041 -Total RJC patients:35,220 Achieved TOO for RJS: 23,376(66%) Not achieved TOO for RJS: 11,844 -Total RC patients: 84,420 Achieved TOO for RS:45,911(54%) Not achieved TOO RS:38,509*
Radical resection	yes	No	yes
No Clavien-Dindo grade ≥ III complications	yes	yes (no 90 days C–D grade ≥ III complications)	no
Length of hospital stay (LOS) ≤ 14 days	yes	yes (LOS ≤ 5 days for CS and ≤ 14 days for RS)	yes (≥ 8 days for non metastasectomy (Stages I–III) patients and ≥ 9 days for patients that received additional metastasectomy)
No 30-day readmission	yes	Yes	yes
No 30-day mortality	yes	No	yes
No conversion to open surgery	yes	No	no
No reintervention	no	Yes	no
No discharge to a rehabilitation/nursing home facility.	no	Yes	no
No postoperative changes in the living situation	no	Yes	no
Lymph node (LN) yield ≥ 12	no	No	yes
Receipt of stage‐appropriate adjuvant chemotherapy	no	No	yes
90-day survival	No	Yes	no

CC, colon cancer; CS, colon surgery.

RC, rectal cancer; RS, rectal surgery.

RJC, rectosigmoid junction cancer; RJS, rectosigmoid junction surgery.

TOO parameters were the same for colon and rectal surgery. The only difference observed between the TOO parameters for colon and rectal cancer was in one study that defined the LOS for both: the LOS for rectal cancer surgery should be ≤14 days, while for colon cancer surgery, it should be ≤5 days (17). In another study, a hospital stay within the 75th percentile of the whole cohort was defined as normal LOS, and it turned out to be ≥8 days for non‐metastasectomy patients and ≥9 days for metastasectomy patients (11). One study mentioned that the LOS should be ≤14 days (18). Two studies included radical resection (11, 18); one study included no Clavien-Dindo grade ≥III complications without a specific time point (18) and another one within 90 days (17). No 30-day readmission and no 30-day mortality were mentioned in two studies (11, 18), while one study mentioned no reintervention and no readmission without a specific time point (17). Most parameters are the same or similar to TOO parameters seen in colon cancer patients.

### Colorectal cancer surgery with no distinction between colon and rectum patients and TOO

We identified three papers that examine textbook outcome in colorectal cancer surgery with no distinction between colon and rectum patients (**Table 4**). A total of 87,421 patients who had colorectal cancer surgery and had registered for TOO were included in the trial. A total of 49,399 patients achieved TOO, while 43,022 did not. All papers were published in 2023. Two of them were multicenter studies (19, 20) while one was a single-center study (21).

**Table 4:** Colorectal Surgery (with no distinction between colon and rectum patients) textbook outcomes.

Article (Setting, period)	Chanza FS et al. (Multicenter, 2004-2015)	Azap L et al. (Multicenter, 2010-2015)	Maeda Y et al. (Single center, 2005-2016)
Patients	*Total*: 40939 *Achieved TOO*: 23580(56%) *Not achieved TOO*: 12359	*Total*: 46296 *Achieved TOO*: 25739(56%) *Not achieved TOO*: 20557	*Total*: 186 *Achieved TOO*: 80(43%) *Not achieved TOO*: 106
No 90‐day mortality.	yes	yes	No
No 90‐day readmission	yes	yes	No
No postoperative complications	yes	yes	No
No extended LOS (beyond the 75th percentile).	yes	yes	No
No 30-day readmission	no	no	yes
Radical resection	no	no	yes
Lymph node (LN) yield ≥ 12	no	no	yes
No Clavien-Dindo grade ≥ II complications in the first 30 days	no	no	yes
No ostomy	no	no	yes
Surgery within 6 weeks	no	no	yes

Two studies had the same TOO definitions: no prolonged LOS beyond the 75th percentile, no 90-day mortality, no 90-day readmission, and no postoperative complications (19, 20). One study included all TOO parameters that had previously been observed in studies involving only colon cancer patients: radical resection, LN yield ≥12, no Clavien-Dindo grade ≥II complications in the first 30 days, no 30-day readmission, and no ostomy (21).

### Factors influencing the achievement of TOO

The characteristics of the TOO and non-TOO groups were compared in a study by Rubio García et al (13). It was found that the non-TOO group had a higher proportion of patients who presented surgical risk and that the TOO group had a significantly higher proportion of females. Differences were also found in the pT classification, with a significantly higher proportion of T3 and T4 and a higher mean number of isolated LNs in the TOO group than in the non-TOO group. Additionally, the laparoscopic approach was more common among TOO patients.

LN yield >12, no stoma, and no adverse outcome were the outcome parameters that most frequently kept patients from reaching a textbook outcome, according to Ching-Chieh Yang et al. (9) Likewise, Yuto Maeda et al. (21) demonstrated the same factors, such as LN yield >12 and absence of adverse events, which resulted in low rates of achieving TOO.

Compared to patients without a textbook outcome, those who achieved TOO had a higher 5-year DSS (9, 14).

Dimitrios K. Manatakis et al. (14) demonstrated factors preventing TOO were older age, left-sided and pT4 cancers. These factors also prevented TOO in another study made by N.E. Kolfschoten et al (7).

In order to evaluate hospital performance, N.E. Kolfschoten et al. utilized TOO, which provides insightful information about the standard of care given to patients with colon cancer and makes it easier to make significant comparisons across different healthcare facilities.

Another work by Ashraf Ganjouei et al. (12) sought to use machine learning methods to provide a decision assistance tool for textbook outcomes. The researchers analyzed data from over 20,000 patients collected from the American College of Surgeons National Surgical Quality Improvement Program database. Patients who achieved a TOO were younger, had lower ASA class, and had an independent functional status compared to those who did not. Following the robotic procedure, TOO was more commonly obtained (76.9%), followed by the laparoscopic procedure (68.3%). Among patients who underwent open colectomy, only 38.8% achieved a TOO. Patients who underwent minimally invasive colectomy had significantly shorter hospital LOS, fewer postoperative complications, lower 30-day readmission rates and lower 30-day mortality rates compared to patients who underwent open colectomy.

Giovanni Taffurelli et al. (17) noted that when minimally invasive surgery, improved recovery protocols, and multidisciplinary management were all used at the same time, most older patients having colorectal cancer surgery could achieve TOO.

According to a study by José Moreira Azevedo et al. (18), robotic colorectal cancer surgery in robotic centers has a high rate of TOO. Even in specialized robotic colorectal facilities, extended resections—like APER—retain a higher chance of failing to reach a TOO in comparison to non-extended resections.

Patients who had a TOO tended to be younger, non-Hispanic White, and more likely to have private insurance, according to Sweigert et al. (15) and Wong et al (11). Individuals who received minimally invasive procedures and had a tumor on the right side were also more likely to have had a TOO. Laparoscopic and robotic techniques were independently linked to increased chances of TOO as compared with open or converted cases. Conversely, there was a correlation found between decreased probabilities of TOO and older age, non-Hispanic Black ethnicity, Hispanic ethnicity, and nonprivate insurance. The likelihood of TOO was also found to be lower in the presence of lymphovascular invasion and increased pathologic tumor stage.

T. Julia T. van Groningen et al. (16) determined TOO’s rankability. The amount of result variation between hospitals that cannot be attributed to random fluctuation is known as rankability. As a result, it might represent the portion of hospital variance attributable to real variations in hospital settings as well as potential variations in care quality. This metric was employed to demonstrate the consistency of hospital rankings based on the particular result. After colon cancer surgery, the rankability of TOO was 54.1%, indicating that about half of the observed differences might be attributed to chance and the other half to the quality of treatment received.

The study by Wong et al. shows that patients treated at safety-net hospitals (SNH), which have a higher proportion of uninsured or Medicaid patients (more than 10%), have a significantly lower likelihood of achieving TOO. Of the 487,195 colorectal cancer patients studied, 66.7% achieved TOO overall. However, those treated at high-burden hospitals (HBH) had an odds ratio (OR) of 0.83 for achieving TOO compared to patients at low-burden hospitals (LBH), reflecting a marked disparity. Key factors affecting TOO achievement at HBHs include the lower rates of adequate lymphadenectomy (87.3%), prolonged LOS (76.3%), and reduced receipt of adjuvant chemotherapy (60.3% for Stage III and 54.1% for Stage IV). Sweigert et al. highlight that insurance status strongly influences the likelihood of achieving TOO. Among the 170,120 patients analyzed, only 54.8% achieved TOO. Patients with private insurance had a higher probability of achieving TOO (OR 1.16) compared to those on Medicaid (OR 0.64) or those uninsured (OR 0.68). This disparity is linked to access to advanced treatments and follow-up care, including timely adjuvant chemotherapy, which was achieved in 83% of the cohort (11)​. Azap et al. show that food insecurity significantly impacts surgical outcomes for colorectal cancer. Among the 46,296 patients who underwent surgery, 20.5% lived in high food insecurity counties. These patients had a 17% higher likelihood of undergoing non-elective surgeries (OR 1.17) and were 11% more likely to experience 90-day readmissions (OR 1.11). High food insecurity patients also had a 32% higher chance of extended hospital stays (OR 1.32) and were 19% less likely to achieve TOO (OR 0.81) compared to patients from low food insecurity counties (20). Shaikh et al. studied 40,939 colorectal cancer patients and found that environmental quality, as measured by the Environmental Quality Index (EQI), is significantly associated with TOO achievement. Patients residing in high EQI counties, which indicate poorer environmental conditions, were 6% less likely to achieve TOO (OR 0.94). This was particularly pronounced among Black patients, who had a 31% lower likelihood (OR 0.69) of achieving TOO when living in moderate-to-high EQI counties compared to White patients in low EQI counties​. Additionally, high EQI areas were associated with higher rates of postoperative complications (21.5%) and extended hospital stays (18.2%), further reducing the chances of achieving an optimal outcome (19).

## Discussion

Thirteen studies covering the topic of TOO in colon and rectal cancer surgery are compiled in this article. Several key themes and findings were identified across the studies. Firstly, the definition of TOO varied among them, but common components included radical resection, LN yield, absence of complications, length of hospital stay, readmissions, and mortality within a specified timeframe. Secondly, our investigation uncovered a consistent link between TOO and enhanced long-term survival outcomes, including both disease-specific survival and overall survival. In fact, compared to patients who did not fulfill TOO requirements, those who did tend to have superior outcomes in terms of survival rates (9, 14).

The detailed analysis of factors influencing the achievement of TOO underlines the complexity of achieving standardized outcomes in colorectal cancer surgery. Some studies introduce unique parameters that could enhance the assessment of TOO. For instance, the use of minimally invasive techniques has been shown to improve TOO achievement (12, 15, 18).

Surgeons are becoming more conscious of their responsibility to let patients know about the standard of treatment they offer. Traditionally, the evaluation of the quality of treatment has been based on discrete measures including duration of stay, distinct resection margin, 30-day readmission, and 30-day mortality (22–24). Nonetheless, patients have indicated a preference for summarized metrics over specific individual outcomes, possibly due to the lack of understanding for some of these individual outcomes (8, 15). In this situation, TOO is not only an important managerial tool, but it also plays a critical role in helping patients make decisions about which health treatments to seek. In order to ascertain the incidence of “true” optimal performance linked to the “ideal” clinical outcome, it provides a clear and easily interpreted statistic (25).

In well-funded institutions, implementing TOO can be more straightforward due to access to advanced surgical techniques, robotic systems, and experienced multidisciplinary teams. These centers should focus on refining TOO standards, ensuring consistent reporting, and using TOO as a metric for continuous quality improvement. In resource-constrained environments, achieving TOO may be more challenging due to limitations in technology and healthcare workforce. However, TOO can still serve as a valuable benchmark for improving outcomes by focusing on attainable goals such as reducing 30-day mortality and readmission rates and minimizing postoperative complications through better surgical planning and patient management. Steps like improving perioperative management, using evidence-based guidelines for colorectal surgery, and implementing enhanced recovery protocols can significantly impact TOO metrics even in less specialized centers. Also, remote consultation with high-volume centers could help surgeons in low-resource settings adopt best practices in colorectal cancer surgery, contributing to achieving TOO. Collaboration between institutions could allow resource-limited hospitals to improve TOO rates by accessing expert advice.

To enhance the clinical utility and universality of the TOO in colorectal cancer surgery, we propose a standardized definition incorporating six essential parameters: radical resection, LN yield ≥ 12, absence of Clavien-Dindo grade ≥ III complications, LOS within the 75th percentile, no 30-day readmissions, and no 30-day mortality. Each parameter plays a critical role in ensuring both the immediate quality and long-term efficacy of surgical oncologic care, as supported by our systematic review.

Ensuring complete resection with negative margins is critical for achieving TOO, as this directly impacts recurrence rates and disease-free survival. This parameter was consistently emphasized across studies, underscoring its significance for favorable prognosis.

Adequate LN retrieval, with a minimum yield of 12 nodes, is a widely accepted oncologic criterion in colorectal cancer surgery. This threshold ensures proper staging, guides adjuvant treatment decisions, and correlates with improved survival outcomes. Studies within our review that included LN yield as a TOO parameter consistently associated it with enhanced survival and more accurate staging.

Severe complications classified as Clavien-Dindo grade III or higher represent significant surgical and postoperative challenges, often leading to reinterventions, extended hospital stays, and increased patient morbidity. The absence of such complications is crucial for TOO. Our review highlights the strong association between lower complication rates and improved long-term outcomes.

A hospital stay within the 75th percentile of similar cases serves as a balanced indicator, promoting optimal recovery without prolonged hospitalization, which can introduce risks such as hospital-acquired infections, patient discomfort, and healthcare costs. Setting the LOS within this percentile not only offers a balanced recovery target but also aligns with enhanced recovery after surgery protocols, promoting optimized care pathways that facilitate safe and efficient discharge, as repeatedly noted in the studies reviewed.

Avoiding readmissions within 30 days post-surgery reflects the effectiveness of discharge planning, postoperative care, and patient education. By including this parameter in TOO, institutions are encouraged to focus on comprehensive discharge protocols and ensure patients receive adequate support post-discharge, minimizing unnecessary hospital utilization. Our review underscores that a lower readmission rate is closely associated with higher patient satisfaction and better long-term recovery, solidifying its place within the TOO definition.

Achieving zero mortality within the first 30 days following surgery is a fundamental criterion for TOO, directly reflecting the quality of surgical and postoperative care. In our review, studies consistently associate lower mortality rates with higher-quality surgical care and better institutional performance, supporting its inclusion as a parameter of TOO.

Together, these six parameters form a comprehensive, standardized definition of TOO that balances surgical quality with patient-centered outcomes, ensuring that high standards are met across different healthcare settings. This TOO definition is flexible enough to accommodate diverse healthcare environments while maintaining rigorous benchmarks, encouraging both resource-rich and limited settings to improve and evaluate their surgical performance based on universally accepted criteria. Standardizing these parameters not only supports consistency in surgical outcome reporting but also promotes comparability across institutions, facilitating advancements in TOO research, enhancing clinical quality measures, and ultimately contributing to improved patient outcomes.

### Limitation

As far as the authors are aware, this is the first systematic review that summarizes TOO in surgery for colon and rectal cancer. It does, however, have some limitations. The retrospective nature of the studies included in this review introduces inherent limitations, such as selection bias and the potential for incomplete or inaccurate data collection. Retrospective studies rely on pre-existing records, which may not consistently capture all relevant patient information, leading to the underreporting of critical variables such as patient comorbidities, nutrition status, or socioeconomic factors. Furthermore, retrospective analyses limit our ability to establish causality between TOO and specific interventions or patient characteristics​. For example, food insecurity and other socioeconomic determinants were not uniformly accounted for in all studies, which may have affected the interpretation of outcomes related to care quality.

Heterogeneity is a limitation in this review, as the included studies vary widely in terms of patient populations, surgical techniques, and institutional settings. Differences in hospital volumes, surgical expertise, and access to resources can all contribute to variability in achieving TOO. For instance, the quality of care in SNHs or institutions serving high-risk populations, such as patients from food-insecure regions, may differ significantly from more resource-rich settings. This variability introduces challenges when trying to draw uniform conclusions about TOO, as the outcomes can be influenced by institutional capabilities, patient demographics, and clinical practices that differ across regions​.

Additionally, patient-level factors such as age, cancer stage, and frailty also contribute to heterogeneity. Studies focusing on elderly populations, for example, reveal that frailty plays a crucial role in the likelihood of achieving TOO​. This variability highlights the difficulty in comparing outcomes across diverse populations without accounting for these individual differences. The generalizability of the findings from this review is limited by the characteristics of the included patient cohorts and healthcare settings. Most of the studies are based on data from high-income countries, where access to advanced surgical techniques and postoperative care is more readily available. Consequently, these results may not be applicable to settings with limited healthcare resources. For example, outcomes from hospitals in low-resource settings, where access to minimally invasive techniques or specialized postoperative care is limited, are underrepresented in this analysis​. Similarly, the findings may not apply to countries or regions with different healthcare structures, such as those where universal healthcare is not available, as factors like insurance status significantly affect TOO.

For these reasons, future studies should stratify TOO outcomes by socioeconomic factors, comorbidities, and surgical techniques to isolate the influence of each confounder. Adoption of a universal TOO definition is essential. Encourage reporting that includes patient-level variables such as SES, comorbidity burden, and surgical approach. Also, ensure that statistical models account for key confounders like age, tumor stage, ethnicity, and hospital type to better reflect the generalizability of TOO.

## Conclusion

Although studies differ in terms of TOO definition and attainment rates, they all concur that one metric is not enough to fully represent the total success of colorectal cancer surgery. Based on the common characteristics identified in the studies included, we propose the adoption of six important factors to formulate a unified definition of TOO for colorectal cancer surgery: radical resection, LN yield ≥ 12, no Clavien-Dindo grade ≥III complications, LOS (75th percentile), no 30-day readmissions, and no 30-day mortality. TOO offers a comprehensive evaluation of surgical outcomes, serving as a valuable metric for optimizing patient care and improving long-term prognosis. It benefits patients in selecting a hospital and provides valuable feedback for healthcare professionals. Also, in low-resource environments, TOO serves as a standardized metric, guiding cost-effective interventions and reducing complications and resource usage.

## Data Availability

The original contributions presented in the study are included in the article/**Supplementary Material**. Further inquiries can be directed to the corresponding author.
