# Supplementary material for: Textbook oncologic outcomes in colorectal cancer surgery: a systematic review
Source: Front Oncol. 2025 May 26;15:1474008. doi: 10.3389/fonc.2025.1474008 (PMC12146897; doi:10.3389/fonc.2025.1474008)
Supplement: Supplementary file 1 [file Table1.docx]

**Table S1.** Quality assessment Newcastle - Ottawa Scale

| Author | Type of study Prospective/retrospective | Selection | | | | Comparability | Outcomes | | | Total score |
| --- | --- | --- | --- | --- | --- | --- | --- | --- | --- | --- |
|  |  | **Representative of the exposed cohort** | **Selection of non-exposed cohort** | **Ascertainment of exposure** | **Outcome of interest not present at the start of the study** | **Comparability of cohorts on the basis of the design or analysis controlled for confounders** | **Assessment of outcomes** | **Sufficient follow-up time** | **Adequacy of follow-up and cohorts** |  |
| Rubio García JJ | Retrospective | ⋆ | ⋆ | ⋆ | ⋆ | ⋆ |  | ⋆ | ⋆ | 7 |
| Yang CC | Retrospective | ⋆ | ⋆ | ⋆ | ⋆ | ⋆ |  | ⋆ | ⋆ | 7 |
| Manatakis DK | Retrospective | ⋆ | ⋆ | ⋆ | ⋆ | ⋆ |  | ⋆ | ⋆ | 7 |
| Kolfschoten NE | Retrospective | ⋆ | ⋆ | ⋆ | ⋆ | ⋆ |  |  |  | 5 |
| Ashraf Ganjouei A | Retrospective | ⋆ | ⋆ | ⋆ | ⋆ | ⋆ |  | ⋆ | ⋆ | 7 |
| Sweigert PJ | Retrospective | ⋆ | ⋆ | ⋆ | ⋆ | ⋆ |  | ⋆ | ⋆ | 7 |
| van Groningen JT | Retrospective | ⋆ | ⋆ | ⋆ | ⋆ | ⋆ |  | ⋆ | ⋆ | 7 |
| Taffurelli G | Retrospective | ⋆ | ⋆ | ⋆ | ⋆ | ⋆ |  | ⋆ | ⋆ | 7 |
| Azevedo JM | Retrospective | ⋆ | ⋆ | ⋆ | ⋆ | ⋆ |  | ⋆ | ⋆ | 7 |
| Wong P | Retrospective | ⋆ | ⋆ | ⋆ | ⋆ | ⋆ |  | ⋆ | ⋆ | 7 |
| Maeda, Y | Retrospective | ⋆ | ⋆ | ⋆ | ⋆ | ⋆ |  | ⋆ | ⋆ | 7 |
| Shaikh CF | Retrospective | ⋆ | ⋆ | ⋆ | ⋆ | ⋆ |  | ⋆ | ⋆ | 7 |
| Azap L | Retrospective | ⋆ | ⋆ | ⋆ | ⋆ | ⋆ |  | ⋆ | ⋆ | 7 |
